# H(2)S-Releasing Aspirin Protects against Aspirin-Induced Gastric Injury via Reducing Oxidative Stress

**DOI:** 10.1371/journal.pone.0046301

**Published:** 2012-09-28

**Authors:** Lei Liu, Jie Cui, Cheng-Jie Song, Jin-Song Bian, Anna Sparatore, Piero Del Soldato, Xin-Yu Wang, Chang-Dong Yan

**Affiliations:** 1 Department of Physiology, Xuzhou Medical College, Xuzhou, Jiangsu, People’s Republic of China; 2 Department of Pharmacology, Yong Loo Lin School of Medicine, National University of Singapore, Singapore, Singapore; 3 Dipartimento di Scienze Farmaceutiche “Pietro Pratesi,” Università degli Studi di Milano, Milan, Italy; 4 CTG Pharma, Milan, Italy; 5 Department of Endocrinology, Shenzhen Second People's Hospital, Guangdong Province, People’s Republic of China; University of Hawaii Cancer Center, United States of America

## Abstract

The aim of this study was to examine the effect of ACS14, a hydrogen sulfide (H_2_S)-releasing derivative of aspirin (Asp), on Asp-induced gastric injury. Gastric hemorrhagic lesions were induced by intragastric administration of Asp (200 mg/kg, suspended in 0.5% carboxymethyl cellulose solutions) in a volume of 1 ml/100 g body weight. ACS14 (1, 5 or 10 mg/kg) was given 30 min before the Asp administration. The total area of gastric erosions, H_2_S concentration and oxidative stress in gastric tissues were measured three hours after administration of Asp. Treatment with Asp (200 mg/kg), but not ACS14 (430 mg/kg, at equimolar doses to 200 mg/kg Asp), for 3 h significantly increased gastric mucosal injury. The damage caused by Asp was reversed by ACS14 at 1–10 mg/kg in a concentration-dependent manner. ACS14 abrogated Asp-induced upregulation of COX-2 expression, but had no effect on the reduced PGE_2_ level. ACS14 reversed the decreased H_2_S concentrations and blood flow in the gastric tissue in Asp-treated rats. Moreover, ACS14 attenuated Asp-suppressed superoxide dismutase-1 (SOD-1) expression and GSH activity, suggesting that ACS14 may stimulate antioxidants in the gastric tissue. ACS14 also obviously inhibited Asp-induced upregulation of protein expression of oxidases including XOD, p47^phox^ and p67^phox^. In conclusion, ACS14 protects Asp induced gastric mucosal injury by inhibiting oxidative stress in the gastric tissue.

## Introduction

Non-steroidal anti-inflammatory drugs (NSAIDs) are among the most commonly prescribed drugs due to their high efficacy in reducing of pain, fever, inflammation and protection against stroke and myocardial infarction [1]. However, their clinical use is commonly associated with the occurrence of adverse effects at the level of digestive tract, ranging from dyspeptic symptoms, gastrointestinal erosions and peptic ulcers to more serious complications, such as overt bleeding or perforation [2]. Redox imbalances appear to play a major pathogenic role in aspirin (Asp) toxicity and gastropathy [3]. To overcome the adverse effects related to NSAID-induced gastrointestinal toxicity, different therapeutic strategies have been evaluated [4]–[6]. This may include reducing the risk of gastrointestinal damage induced by NSAIDs and enhancing the protective function of the gastric mucosa.

Hydrogen sulfide (H_2_S) is now well recognized to be an endogenous gaseous mediator. Like nitric oxide, another “gasotransmitter”, H_2_S also regulates various physiological functions [7], [8]. It was recently found that H_2_S produces strong anti-oxidative [9], anti-apoptotic [10] and anti-inflammatory effects [11] in different tissue injuries. H_2_S and H_2_S-releasing molecules are able to enhance intracellular antioxidant activities by means of several mechanisms, including stimulation of glutathione and induction of the antioxidant and tissue protective protein heme oxygenase-1 [12]–[14].

ACS14 is a H_2_S releasing compound, 2-acetyloxybenzoic acid 4-(3-thioxo-3*H*-1, 2-dithiol-5-yl) phenyl ester (ACS 14, S-aspirin) [15], [16]. The pharmacological profile of ACS14 was described recently [15]. It contains a dithiolethione moiety which gradually releases H_2_S for a sustained period [17]. In the present study, we therefore investigated the effect of ACS14 on Asp-induced gastric mucosal injury by examining whether ACS14 can prevent Asp-induced redox imbalances in rats.

## Methods

### Animals

Male Sprague-Dawley rats, 200–240 g, were obtained from the Animal Center of Xuzhou Medicine College (Xuzhou, China) and were housed at 22°C in a controlled environment with 12 h of artificial light per day. They were fasted for 20–24 h before the experiments but had free access to drinking water. All animal experiments were conducted in accordance with international ethical guidelines and the experimental protocols for using rats have been reviewed and approved by the Animal Ethics Committee at Xuzhou Medicine College.

### Asp-induced Gastric Mucosal Injury and ACS14 Treatment

Gastric hemorrhagic lesions were induced by intragastric administration of Asp (200 mg/kg in 0.5% carboxymethyl cellulose solutions) in a volume of 1 ml/100 g body weight. To investigate the preventive effect of ACS14 on Asp-induced gastric mucosal injury, ACS14 synthesized as previously described [15] at doses of 1, 5 or 10 mg/kg (dissolved in DMSO) was injected intraperitoneally 30 min before the administration of Asp. Three hours after administration of Asp, the animals were killed by over-dose injection of pentobarbital sodium (60 mg/kg i.p.) and stomachs were harvested for other experiments.

### Gastric Damage: Macroscopic Analysis

Both cardia and pylorus of stomach were ligated. 10 ml of 10% formaldehyde solution was injected into gastric cavity. The whole stomach was fixed in the same concentration of formaldehyde solution overnight. On the second day, the stomach was opened along the greater curvature, washed lightly and flattened on a piece of cardboard. The total number of gross mucosal lesions per stomach was counted and each lesion was scored according to the following scheme: grade1: petechial lesion, grade 2: lesion≤2 mm, grade 3: 2< lesion≤4 mm, grade 4: 4< lesion≤6mm and grade 5: lesion greater than 6 mm.

### Measurement of H_2_S Concentration in Plasma and Gastric Tissue

The method for measurement of H_2_S concentration was described in our previous publications [18], [19]. Briefly, 75 µl plasma or gastric mucosal homogenates from each group were diluted in deionized water (final volume, 500 µl). H_2_S was trapped by addition of zinc acetate (1% w/v, 250 µl). Subsequently, N, N-dimethyl-p-phenylenediamine sulphate (20 µM; 133 µl) in 7.2 M HCl was added, followed by FeCl_3_ (30 µM; 133 µl) in 1.2 M HCl. Thereafter, trichloroacetic acid (10% w/v, 250 µl) was used to precipitate any protein that might be present in the culture media and upon centrifugation (10,000 g) absorbance (670 nm) of aliquots from the resulting supernatant (300 µl) was determined using a 96 well microplate reader [20].

### Determination of PGE_2_ Levels

Tissue from each rat stomach was removed, weighed (approximately 0.1 g), and placed in a test tube containing 1 ml of 0.1 M phosphate buffer, pH 7.4, 1 mM EDTA, and 10 µM indomethacin. The tissue was homogenized and centrifuged for 20 min at 1,000 g at 4°C. Prostaglandin E_2_ (PGE_2_) content in supernatant was determined by an enzyme immunoassay kit following the protocol described by the manufacturer (Biovol Technologies, China). Results are expressed as picograms of PGE_2_ per milligram of protein. Proteins were determined by using the bicinchoninic acid (BCA) kit (Beyotime Institute of Biotechnology, China).

### Measurement of Malondialdehyde (MDA) Levels and Glutathione (GSH) Activity in Gastric Tissue

Approximately 0.5 g of gastric tissue from individual rats was homogenized in 4.5 ml physiological saline and the supernatants were obtained by centrifugation at 2,000 g for 10 min. The protein concentration in the gastric mucosal homogenates was measured by using the bicinchoninic acid (BCA) kit (Beyotime Institute of Biotechnology, China). MDA levels and GSH activity in gastric tissue supernatants were measured using the enzyme-specific activity detection kits (Nanjing Jiancheng Bioengineering Co., China), according to the manufacturer’s instructions.

### Determination of Gastric Blood Flow

Rats were anesthetized with pentobarbital sodium (60 mg/kg i.p.) and operated along the mid-line of abdomen to expose the stomach. Laser Doppler blood flow meter and miniature surface probes (moorVMS, UK) were used to record the blood flow. The acquired signal was converted to blood perfusion unit (BPU) and recorded with a computer. The curve was analyzed with moorVMS v1.0 software. Only stable signals were included and calculated. Blood flow was recorded for three times (15 s for each time) in each rat.

### Western Blot Analysis

Gastric samples were lysed in buffer. The protein concentration of each lysate was determined using the BCA kit according to the manufacture's protocol. 7.5%, 10% or 12.5% SDS-polyacrylamide gels were used depending on the molecular weight of the measured proteins. After electrophoresis, the polyvinylidene fluoride (PVDF) membranes were washed in Tris-buffered saline containing 0.1% Tween-20 (TBST) for 1 h, and then incubated with the relevant antibody at 4°C overnight. All antibodies (anti-SOD1 antibody, anti-XOD antibody, anti-COX2 antibody, anti-phosphorylated p22^phox^ antibody, anti-p47^phox^ antibody, anti-p67^phox^ antibody and anti-gp91^phox^ antibody) were purchased from Santa Cruz Biotechnology Inc., Santa Cruz, CA, USA. Membranes were washed three times in TBST buffer (10 mmol/l Tris, pH 7.5; 150 mmol/l NaCl, 0.05% Tween-20), followed by incubation with secondary antibody. The NBT/BCIP western blot analysis system according to the manufacturer's protocol was used for detection the protein signals. The results are the average of four independent experiments.

### Statistical Analysis

All data were presented as mean ± SEM. Statistical significance was assessed with one-way analysis of variance (ANOVA) followed by a post hoc (Bonferroni) test for multiple group comparison. Differences with p-value less than 0.05 were considered statistically significant.

## Results

### Effect of ACS14 and Asp on Gastric Mucosa

**Figure 1:**
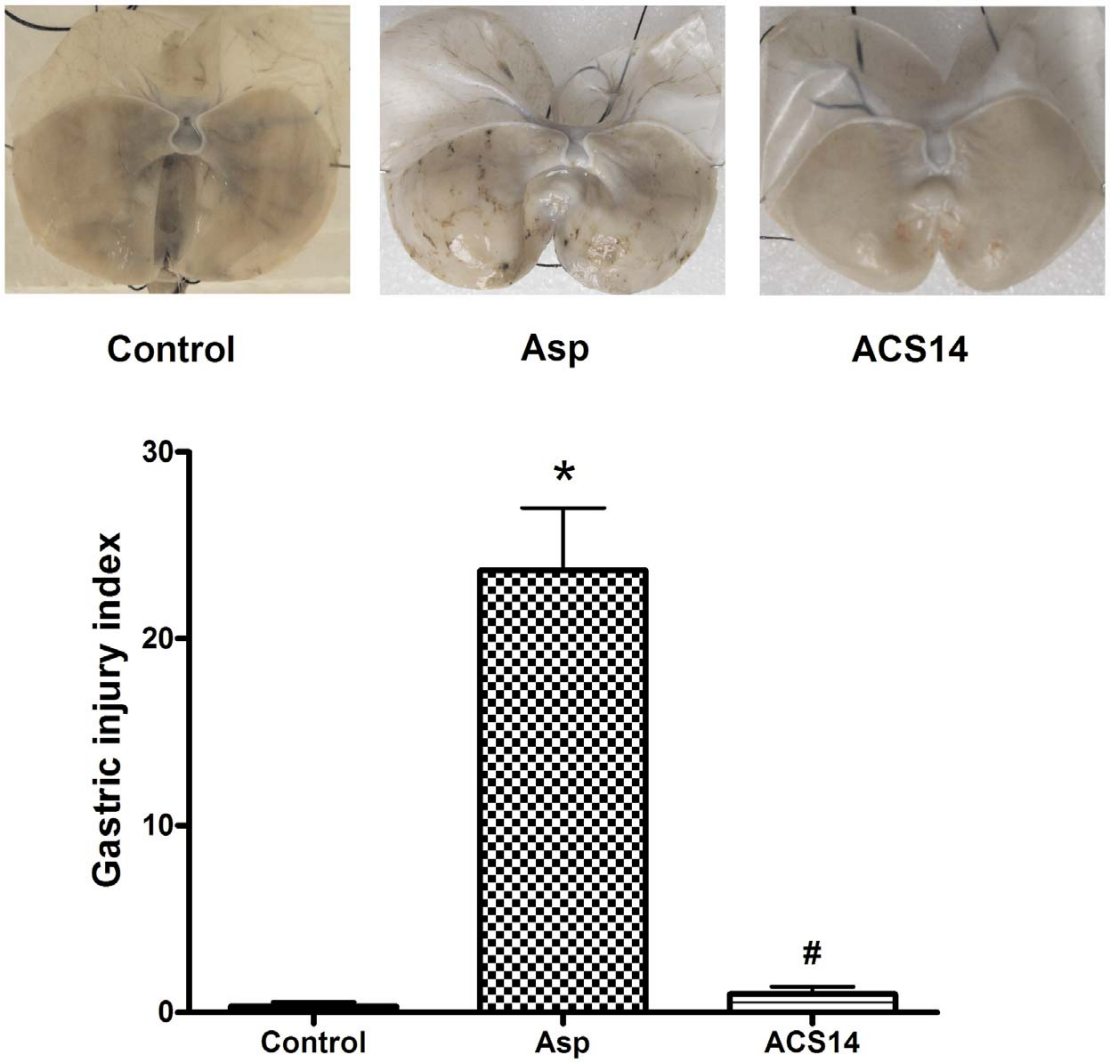
Effect of Asp at 200 mg/kg and ACS14 (430mg/kg) on the morphology of gastric mucosa in rats. ACS14 or Asp was administered to rats 3 h by intragastric administration. Representative photographs (A) and group data (B) showing that Asp, but not ACS14, induced significant gastric mucosal injury. Data are presented as means ± SE. n = 6. * P<0.05 compared with control; # P<0.05 compared with Asp.

We first compared the effect of ACS14 and Asp on gastric mucosa. We found that intragastric administration of ACS14 at 430 mg/kg (at equimolar doses to 200 mg/kg Asp) did not cause any damage in the gastric mucosa. However, Asp at 200 mg/kg induced severe mucosal damage. These data suggest that H_2_S released from ACS14 may protect against Asp-induced gastric damage (Fig. 1).

### Effect of ACS14 and NaHS on Asp-induced Gastric Mucosal Injury

**Figure 2:**
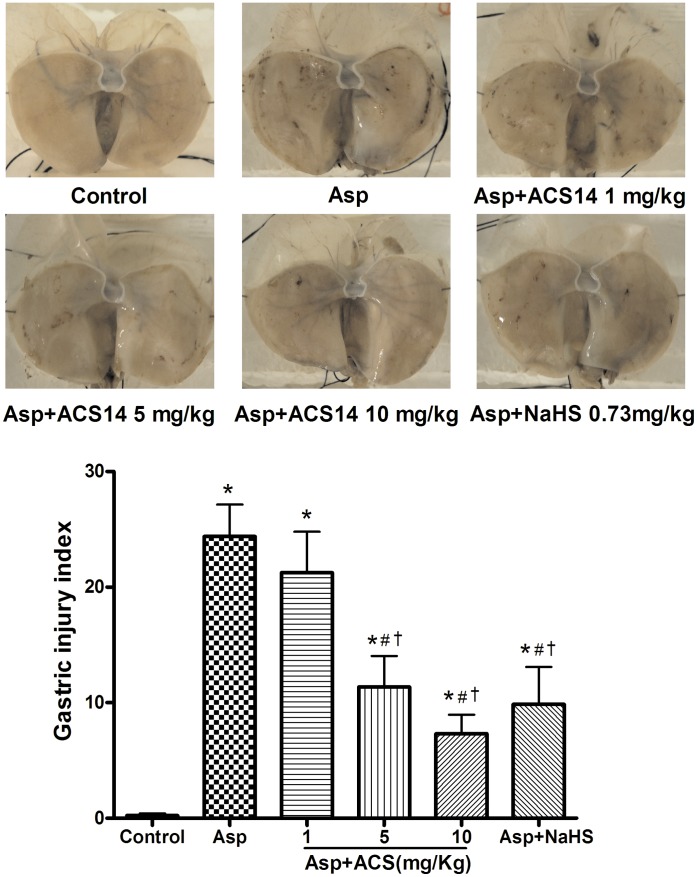
Effect of ACS14 on Asp-induced gastric mucosal injury in rats. ACS14 (1, 5 or 10 mg/kg) was given (i.p.) 30 min before intragastric administration of Asp (200 mg/kg). Representative photographs (A) and group data (B) showing ACS14 significantly attenuated Asp-induced gastric mucosal injury. Data are presented as means ± SE. n = 8. *P<0.05 compared with control; #P<0.05 compared with Asp; †P<0.05 compared with Asp+ACS14 1 mg/kg.

Treatment of rats with Asp (200 mg/kg) for 3 h significantly increased gastric mucosal injury. As shown in Fig. 2, Asp induced the appearance of multiple visible gastric petechial erosions. The size of erosions ranged from 2 to 10 mm in length and about 1 mm in width. This damage was reversed by ACS14 at 1–10 mg/kg in a concentration-dependent manner. The significant effect was observed when ACS14 was at 5–10 mg/kg. NaHS (an H_2_S donor) at 0.73 mg/kg (produced H_2_S approximately equivalent to that caused by ACS14 at 5 mg/kg) also decreased the gastric mucosal injury induced by Asp to a similar extent caused by ACS14 at 5 mg/kg. These data imply that ACS14 may protect gastric mucosa against Asp-induced mucosal injury via releasing H_2_S.

### ACS14 and NaHS Increased H_2_S Concentrations in Plasma and Gastric Tissue

**Figure 3:**
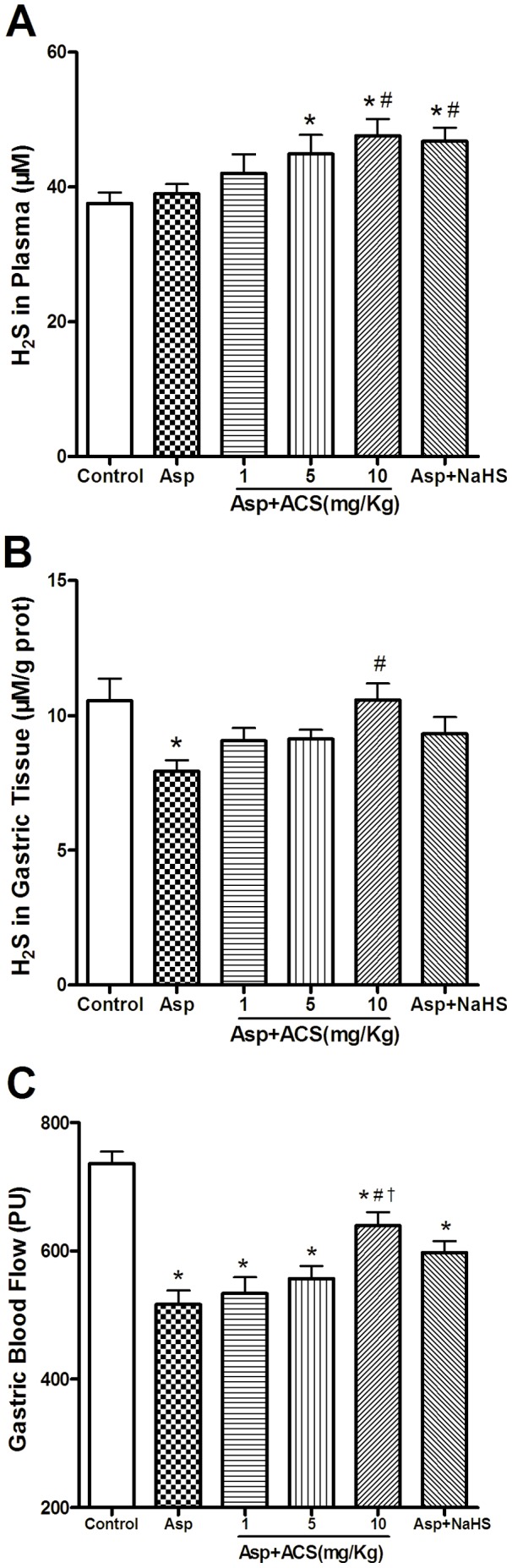
Effect of ACS14 on H_2_S concentration and basal gastric mucosal blood flow in rats. A & B. ACS14 increased H_2_S concentrations in both blood plasma (A) and gastric tissues (B). n = 7. C. ACS14 increased basal gastric mucosal blood flow in rats. n = 8. Data are the means±SE. *P<0.05 compared with control; #P<0.05 compared with Asp; †P<0.05 compared with Asp+ACS14 1 mg/kg.

Rats treated with Asp didn’t affect the H_2_S concentrations in plasma. Treatment with ACS14 (5 and 10 mg/kg) and NaHS (0.73 mg/kg) significantly increased the H_2_S concentrations in plasma (Fig. 3A). Interestingly, Asp treatment significantly decreased the local H_2_S concentration in gastric tissue. This effect was reversed by ACS14 pretreatment at 10 mg/kg. (Fig. 3B).

### Effect of ACS14 and NaHS on Gastric Blood Flow

Since H_2_S may dilate blood vessel, we therefore examined the effect of ACS14 on gastric blood flow. As shown in Fig. 3C, Asp significantly decreased gastric blood flow. This effect was reversed by ACS14 at 10 mg/kg. ACS14 at 1 mg/kg, 5 mg/kg and NaHS at 0.73 mg/kg failed to significantly change the gastric blood flow.

### Effects of ACS14 on COX-2 Expression and PGE_2_ Content in Gastric Tissue

**Figure 4:**
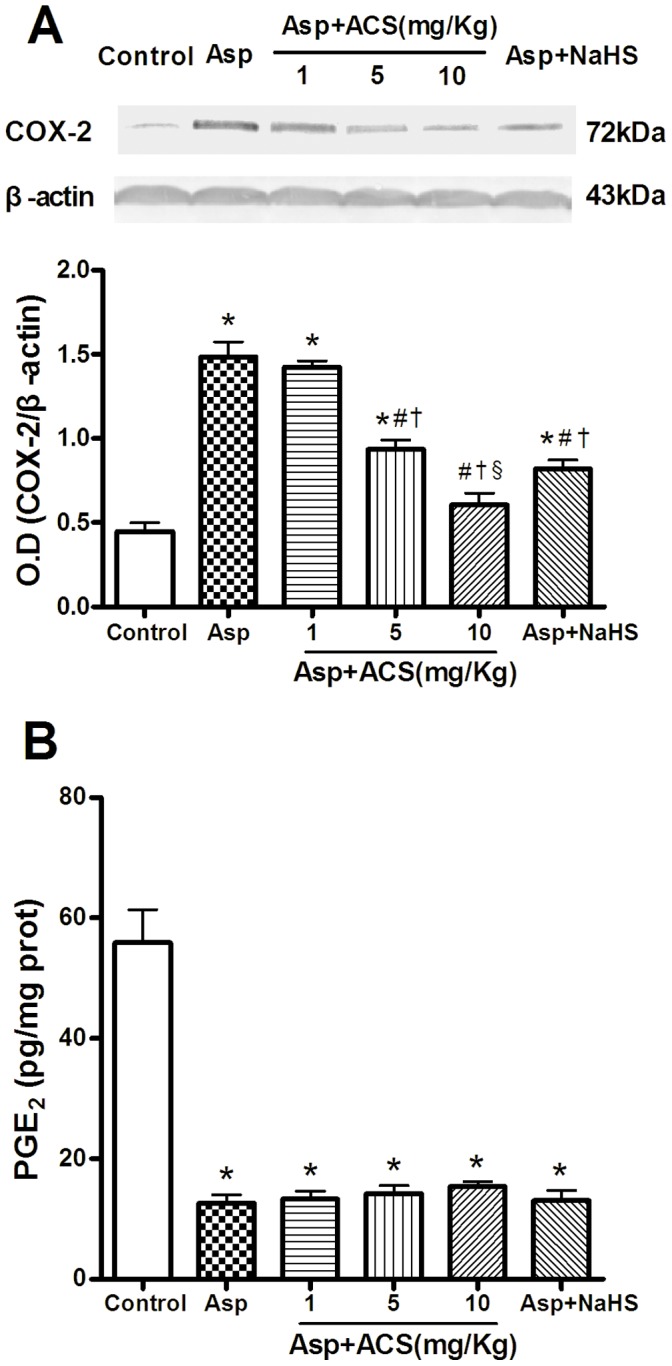
Effect of ACS14 on COX-2 expression (A) and PGE_2_ content (B) in gastric tissue. A. Representative Western blots and group data showing that ACS14 reversed Asp-upregulated COX-2 expression. n = 4. B. Both ACS14 and NaHS failed to change Asp-suppressed PGE_2_ level in gastric tissue. n = 7. Data are the means ± SE.*P<0.05 compared with control; #P<0.05 compared with Asp; †P<0.05 compared with Asp+ACS14 1 mg/kg; §P<0.05 compared with Asp+ACS14 5 mg/kg.

Cyclooxygenase 2 (COX-2) is an inducible enzyme that participates in inflammation by producing prostanoids including PGE_2_. COX-2 expression was significantly increased in the gastric tissue after treatment with Asp (Fig. 4A). Pretreatment with ACS14 at 5–10 mg/kg and NaHS at 0.73 mg/kg reversed the up-regulated expression of COX-2, suggesting that the protective effects may be mediated by suppression of COX-2 expression. However, the PGE_2_ level was markedly reduced in rats treated with Asp (200 mg/kg). This effect was not rescued by treatment with either ACS14 or NaHS (Fig. 4B).

### ACS14 and NaHS Decreased Asp-induced Gastric Oxidative Stress

**Figure 5:**
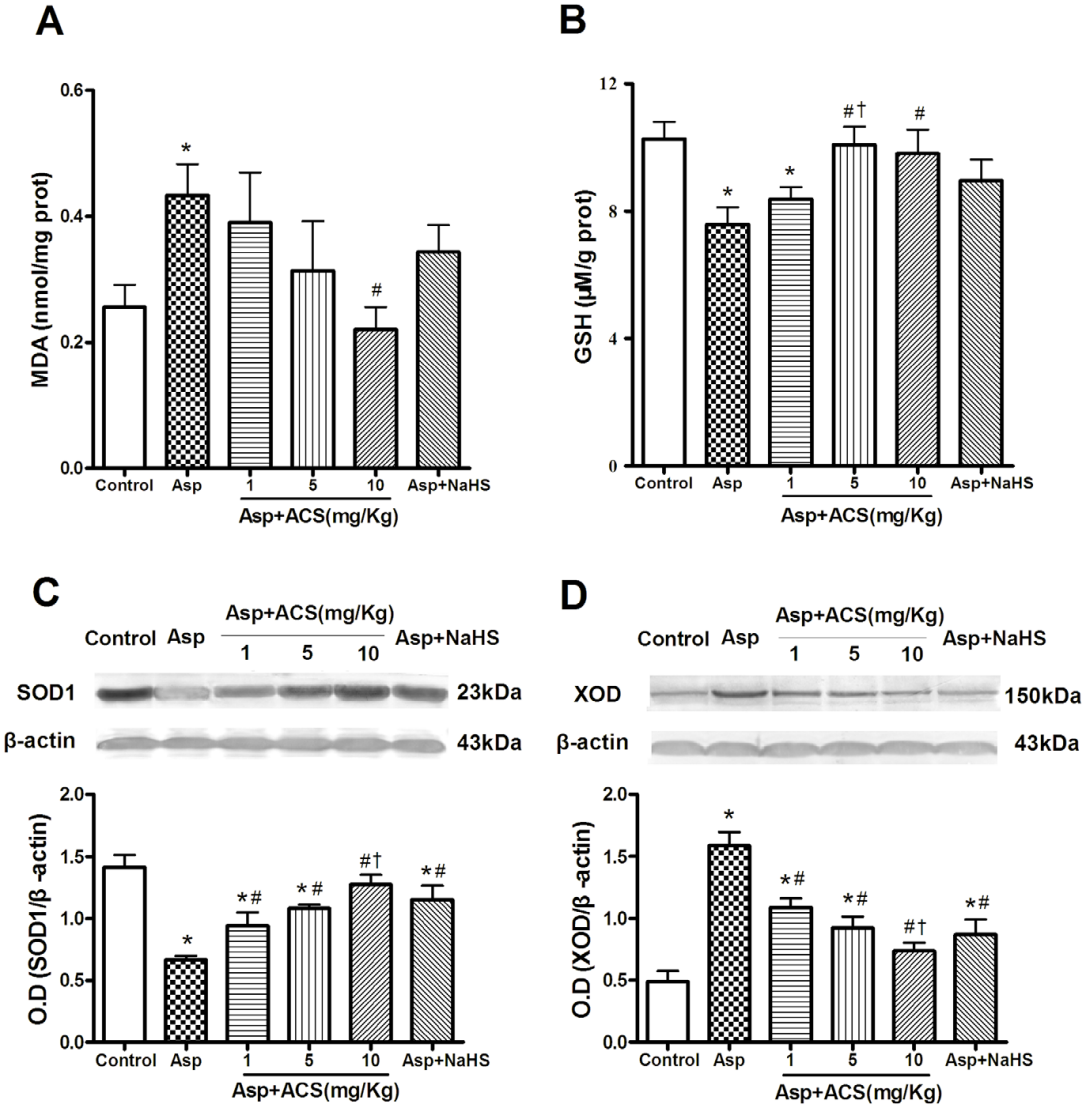
Effect of ACS14 on levels of MDA (A) and GSH (B) and protein expressions of SOD1 (C) and XOD (D) of gastric tissues in Asp-treated rats. Data are presented as means±SE. n = 4–8. *P<0.05 compared with control; #P<0.05 compared with Asp; †P<0.05 compared with Asp+ACS14 1 mg/kg.

It is well known that Asp-induced gastric injury is caused by oxidative stress [3], [21]. We therefore examined the levels of gastric MDA, one of the markers of free radical species-related injury. As expected, the levels of gastric MDA were significantly elevated in Asp group of rats, as compared with the control group (Fig. 5A, P<0.05). Pretreatment with ACS14 at 10 mg/kg obviously reduced the elevated MDA level. Treatment with NaHS (0.73 mg/kg) showed a trend of decreasing gastric MDA when compared with Asp alone group, but no significant difference was found. The results suggest that ACS14 appeared to be a potent antioxidant regulator to attenuate the Asp-induced gastric injury in rats.

GSH is the most important antioxidant. As shown in Fig. 5B, Asp markedly suppressed intracellular GSH production from 10.27±0.54 µmol/g protein to 7.59±1.54 µmol/g protein. This is consistent with the previous findings [22]. Pretreatment with ACS14 at 5 and 10 mg/kg significantly increased the gastric GSH level.

SOD-1 is one of three superoxide dismutases responsible for destroying free superoxide radicals in the body. As shown in Fig. 5C, Asp treatment significantly decreased the expression of SOD-1 in the gastric tissue. Similarly, pretreatment with ACS14 and NaHS reversed the down-regulation of SOD-1 expression induced by Asp.

Xanthine oxidase (XOD) is an oxidase which produces reactive oxygen species. XOD expression in gastric tissue was significantly increased by Asp treatment (Fig. 5D). Treatment with ACS14 and NaHS suppressed Asp-induced upregulation of XOD expression.

**Figure 6:**
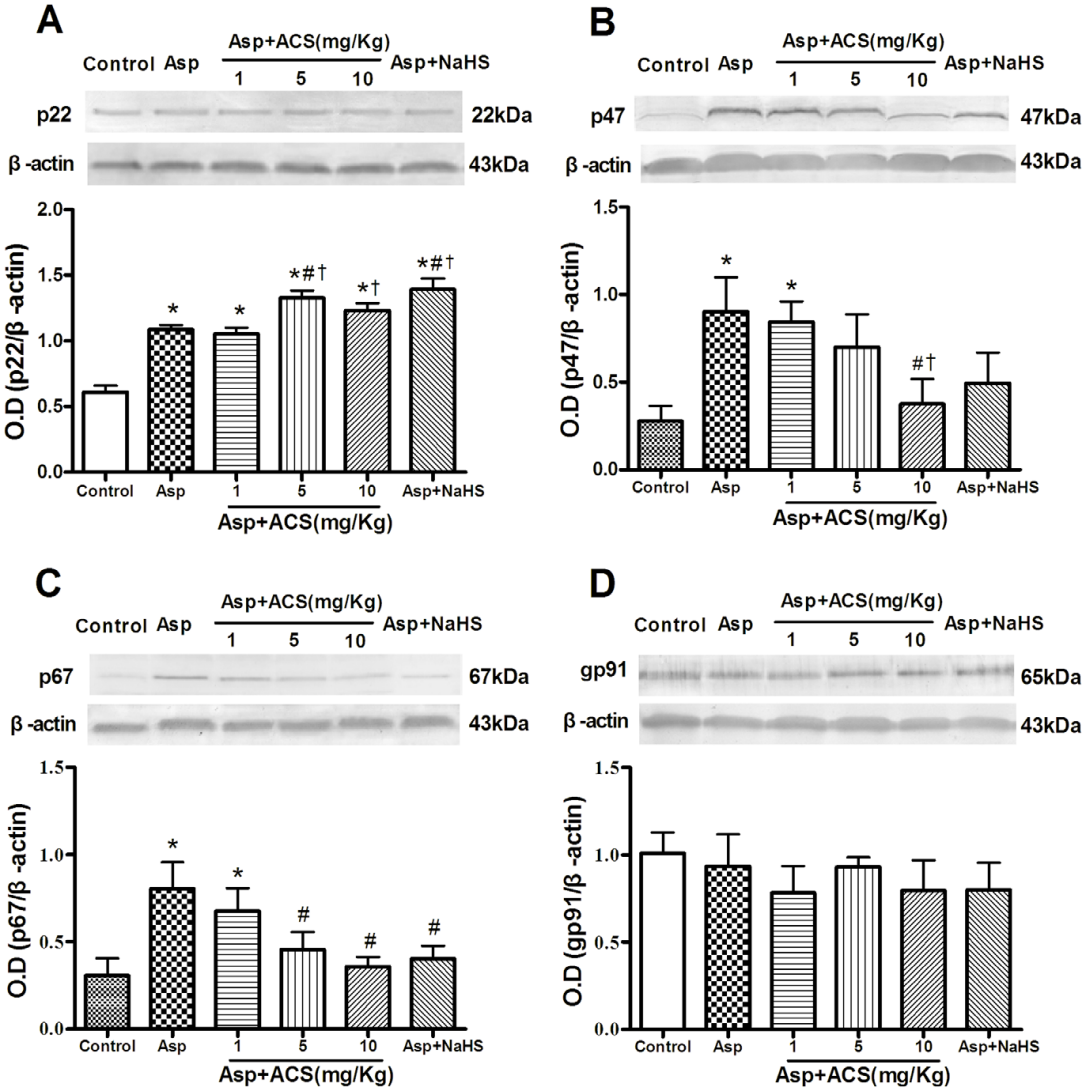
Effect of ACS14 on protein expressions of p22^phox^, p47^phox^, p67^phox^ and gp91^phox^ protein expressions of gastric tissue in Asp-treated rats. Data are presented as means ± SE. n = 4. *P<0.05 compared with control; #P<0.05 compared with Asp; †P<0.05 compared with Asp+ACS14 1 mg/kg.

The NADPH oxidase is a membrane-bound enzyme complex. It generates superoxide by transferring electrons from NADPH to molecular oxygen to produce the superoxide. NADPH comprises p22^phox,^ gp91^phox^, p40^phox^, p47^phox^, p67^phox^, and the small GTP-binding protein Rac [23]–[25]. As shown in Fig. 6, Asp significantly upregulated the protein expression of p22^phox^ (Fig. 6A), p47^phox^ (Fig. 6B) and p67^phox^ (Fig. 6C), pretreatment with ACS14 and NaHS significantly attenuated the expression of p47^phox^ and p67^phox^, but enhanced the expression of p22^phox^. However, all the three drugs, Asp, ACS14 and NaHS had no significant effect on gp91^phox^ (Fig. 6D).

## Discussion

Asp is widely used as an anti-inflammatory and analgesic drug. However, Asp at therapeutic dose for pain-relief often induces gastrointestinal adverse effects including gastric ulcer and bleeding. At low doses, Asp is also used to prevent cardiovascular and cerebrovascular disease [26], [27]. The recent clinical studies showed that even at low dose to prevent cardiovascular disease. Asp also induces gastroduodenal complications [28]–[31]. The annoying adverse effect may largely limit the clinical uses of NSAIDs. Therefore, development of new salicylate drugs which may not produce gastrointestinal toxicity is necessary and urgent.

H_2_S is increasingly being recognized as a fundamental signaling molecule, and many H_2_S-releasing compounds were developed in recent years [15], [32]–[34], such as H_2_S-releasing naproxen and H_2_S-releasing Asp. It has recently been found that NaHS significantly attenuated the gastric damage caused by Asp [31]. This prompted us to investigate whether H_2_S releasing Asp can still produce gastric injury. ACS14 is a developed H_2_S-releasing Asp. We found in the present study that ASC14 at the same dose as Asp failed to produce gastric injury, suggesting that H_2_S released from ACS14 may protect stomach against Asp-induced injury.

We then moved on to study whether ACS14 at low doses can prevent harmful effect of Asp in stomach. We found that ACS14 at 1–10 mg/kg reversed Asp induced gastric damage in a concentration-dependent manner. ACS14 is known to release H_2_S in vitro and in vivo [15], [35], [36]. We also found in the present study that ACS14 (at 5 and 10 mg/kg) significantly increased the H_2_S concentrations in plasma. These data confirmed that the beneficial effect was from the released H_2_S from ACS14.

COX is the rate-limiting enzyme to regulate the synthesis of prostaglandins by conversion of arachidonic acid to PGH_2_, the common precursor of bioactive prostaglandins. Two distinct COX isoforms were reported. COX-1 is responsible for constitutive prostaglandin formation, whereas COX-2 is usually induced in response to stress [37]. It was reported that Asp can rapidly up-regulate COX-2 expression in the stomach [38]–[40]. We found in the present study that ACS14 at 1–10 mg/kg reversed the up-regulated expression of COX-2, in a dose-dependent manner. This is consistent with a previous study that H_2_S significantly attenuated Asp-induced upregulation of COX-2 mRNA level [39].

Endogenous PGE_2_ derived from COX-2 is closely related to the recovery of gastric mucosal injury [41], [42] and plays an important role for the maintenance of gastric mucosal integrity by preventing exogenous injury to the stomach and accelerating gastric mucosal healing [43]. It was found in the present study that Asp markedly decreased PGE_2_ production. We therefore proposed that the upregulated COX-2 produce level was secondary to a compensatory response to inhibition of COX-2 activity and gastrin PG synthesis [38]. However, we found that neither ACS14 nor NaHS reversed Asp-impaired PGE_2_ production. Our data suggest that the protective effects of ACS14 and NaHS were not mediated by PGE_2_.

Oxidative stress is associated with increased production of oxidizing species or a significant decrease in the capability of antioxidant defenses [44]. H_2_S scavenges oxygen-derived free radicals [9], [45]–[49], which mediates the protective effects of NaHS against the toxicity of H_2_O_2_ in cells in vitro and also the ischemia-reperfusion-induced gastric mucosal damage in rats in vivo [47], [50]. We found in the present study that ACS14 significantly reduced Asp-induced elevation of MDA, one of the markers of free radical species-related injury. Glutathione is the major cellular antioxidant and plays an important role in antioxidative stress by H_2_S [9], [35], [51]–[53]. H_2_S protects neurons from oxidative stress by increasing the levels of GSH [9], [51], [54]. We found in the present study that ACS14 significantly increased the gastric GSH level. In addition, ACS14 also reversed Asp-reduced protein expression of SOD, which is responsible for converting superoxide radicals to molecular oxygen and hydrogen peroxide within cytoplasm and mitochondria [55]. Our data suggest that ACS14 may protect the gastric mucosa against Asp-induced damage via upregulation of antioxidants level.

We also examined the expressions of redox enzymes, NADPH oxidase. NADPH oxidase is a multicomponent enzyme that comprises p22^phox,^ gp91^phox^, p40^phox^, p47^phox^, p67^phox^, and the small GTP-binding protein Rac [23]–[25], [56]. We found in the present study that Asp significantly upregulated the protein expression levels of 22^phox^, p47^phox^ and p67^phox^, but not that of gp91. These data suggest that Asp may activate NADPH oxidase by stimulating some subunits of the complex. ACS14 at 10 mg/kg obviously attenuated Asp-induced upregulation of p47^phox^ and p67^phox^ subunit expression and therefore protected gastric tissue. Although p22^phox^ expression was further increased by ACS14 and NaHS, which didn’t influence the protective role on Asp-induced gastric injury. This is consistent with the previous findings that NaHS can inhibit NADPH oxidase expression and concomitant O_2_.^−^ formation [25], [57]–[59].

XOD catalyzes the conversion reactions of hypoxanthine to xanthine and xanthine to uric acid, the last reaction in the purine catabolism, with byproduct of toxic superoxide radical. In this regard, it is a key enzyme between purine and free radical metabolism [60]. It was reported that XOD is an endogenous source of ROS and reactive nitrogen species (RNS) that can induce oxidative stress and inflect tissue injury [61]. Our findings showed that Asp significant increased XOD protein level in gastric tissue and this effect was reversed by ACS14. Taken together, our data clearly demonstrated that ACS14 may protect gastric mucosa by suppression of oxidative stress.

We also investigated the effect of ACS14 on the gastric blood flow. It was found that ACS14 obviously increased Asp-reduced gastric blood flow. This may further contribute to its anti-oxidant effect as sufficient blood flow and oxygen supply may wash-out/inhibit Asp-induced O_2_.^−^ production in gastric tissue. The mechanism underlying the ACS14-increased gastric blood flow may involve opening of K_ATP_ channels. This is supported by a previous study which showed that systemically application of exogenous H_2_S increased gastric mucosal blood flow by activation of K_ATP_ channels [50].

In conclusion, we demonstrated in the present study ACS14, an H_2_S releasing Asp, protects gastric mucosa against Asp induced injury via inhibition of oxidative stress and increasing blood flow locally.
